# LoRa Network-Based System for Monitoring the Agricultural Sector in Andean Areas: Case Study Ecuador

**DOI:** 10.3390/s22186743

**Published:** 2022-09-07

**Authors:** Edgar Fabián Rivera Guzmán, Edison David Mañay Chochos, Mauricio Danilo Chiliquinga Malliquinga, Paúl Francisco Baldeón Egas, Renato Mauricio Toasa Guachi

**Affiliations:** 1 Instituto Superior Universitario Tecnológico Oriente, La Joya de los Sachas 220101, Ecuador; 2 Alfa Soluciones—Ingeniería, Salcedo 050550, Ecuador; 3 Departamento de Ciencias de la Ingeniería, Universidad Tecnológica Israel, Quito 170516, Ecuador

**Keywords:** Andean region, intelligent agriculture, LoRa technology, low-cost LoRa node and gateway, wireless sensor networks, IoT system

## Abstract

This article focuses on the development of a system based on the long-range network (LoRa), which is used for monitoring the agricultural sector and is implemented in areas of the Andean region of Ecuador. The LoRa network is applied for the analysis of climatic parameters by monitoring temperature, relative humidity, soil moisture and ultraviolet radiation. It consists of two transmitter nodes and one receiver node, a LoRa Gateway with two communication channels for data reception and one for data transmission, and an IoT server. In addition, a graphical user interface has been developed in Thinger.io to monitor the crops and remotely control the actuators. The research conducted contains useful information for the deployment of a LoRa network in agricultural crops located in mountainous areas above 2910 m.a.s.l., where there are terrains with irregular orography, reaching a coverage of 50 hectares and a range distance of 875 m to the farthest point in the community of Chirinche Bajo, Ecuador. An average RSSI of the radio link of −122 dBm was obtained in areas with a 15% slope and 130 m difference in height according to the Gateway, where the presence of vegetation, eucalyptus trees and no line-of-sight generated interference to the radio signal. The success rate of PDR packet delivery with an SF of nine, had a better performance, with values of no less than 76% and 92% in uplink and downlink respectively. Finally, the technological gap is reduced, since the network reaches places where traditional technologies do not exist, allowing farmers to make timely decisions in the production process in the face of adverse weather events.

## 1. Introduction

### 1.1. Motivation

Great technological progress directly affects people’s behavior. Humans have adapted themselves to the use of the Internet, computer systems, smartphones and sensors, among other devices to carry out different daily activities. In the same way, different types of applications have been developed to meet our needs, allowing problem solving in many areas of interest. One of the main areas that has benefited from technology is the agricultural sector. According to the Food and Agriculture Organization of the United Nations (FAO), the world should produce 70% more food in 2050 than what it produced in 2006 to feed the growing population around the world [1].

The Agricultural sector is turning to the IoT for analysis, management and the search for greater production capacities as the demands for the products and its operation increase. The growth of communication technologies and sensors for agriculture, their easy usage and operation, as well as their low production cost, allow different parameters such as humidity, temperature and soil acidity to be measured in different places [2]. They can even be used in regions of difficult access, such as mountainous areas, slopes and deserts, and other possible places where agriculture can be developed.

In this regard, the use of these technologies allows farmers to analyze the data collected by the sensors, predict future climatic conditions and, therefore, improve productivity, minimize expenses and preserve the resources used.

### 1.2. Problem Definition and Contribution

In Ecuador, the use of intelligent sensors at the agricultural level is very limited. Currently, there are sensors that are used for census, monitoring and control of multiple variables [3]. The use of these wireless sensors allows for efficient energy management. They are scalable. New sensors can be incorporated without affecting performance, achieving the implementation of dynamic network topologies [4].

The contribution of this work is to reduce the technological gap in rural communities in Ecuador, which do not have many economic resources, and with this network, farmers will be able to control production and pests, based on the climatic changes present in the area.

For the coverage area where the network is implemented, the applied system offers scalability, security, management and an affordable cost for the community, whose economic income depends mostly on agricultural activities. In this context, the system will serve as a basis for the implementation of techniques such as phytosanitary control, intelligent irrigation, and production forecasting among others.

## 2. Related Works

Extensive research was conducted on the LoRa network and communication technologies. Foremost, it is identified that Low-Power Wide-Area (LPWAN) technologies have become popular worldwide [5]; such as LTE-M [6], SigFox, Narrow Band (NB)-IoT [7] and long-range LoRa [8]. The latter two being the ones that dominate wireless communications.

Comparing these two technologies, it was identified that LoRa has advantages in terms of battery life, its capacity and cost; while NB-IoT offers benefits in terms of service quality (QoS), latency, reliability and range [9,10]. It was also pointed out that despite the extensive research that has been conducted so far on existing LPWAN technologies, there are still challenges to be addressed that can be of great assistance to the scientific and academic community.

Regarding the use of LoRa in agriculture, [11] proposes the development of a mobile gateway device with low-power wide-area networks (LoRaWAN), used to increase the productivity and efficiency of greenhouses. The results showed that this approach helps current agricultural processes, due to the low cost and accuracy of humidity and temperature measurements. On the other hand, in [12], a system was developed to transmit uninterrupted images taken from a camera in a static environment through LoRa, the purpose of which was to reduce the amount of data transmitted while maintaining the quality of the image and service.

Research on agricultural monitoring with LoRa networks was also conducted. In [13], a soil environment monitoring system based on a radio frequency identification (RFID) sensor and LoRa is presented to conduct long-term and low-cost monitoring. In addition, in [14], a long-range, low-power IoT network was recommended to monitor soil moisture; using LoRa as the communication interface, which uses the 868 MHz ISM band for signal transmission. On the other hand, a model called AgriPrediction [15] was presented, which combines a short and medium-range wireless network system with a prediction engine to proactively predict changes in crops, thus notifying the farmer of corrective actions. Finally, studies on the state-of-the-art and the current situation in agriculture, IoT and the LoRa network [16,17,18] were reviewed. These studies represent a great starting point to arrive at intelligent agriculture.

Other studies referring to the use of IoT in agriculture were identified in [19]. They developed an object detection model for the monitoring and control of weeds in grasslands in the state of California. After a series of tests, the model showed an average of 94% accuracy in image detection, which is better than other models identified in the literature. On the other hand, in [20], a software framework based on a fuzzy logic system for the evaluation and cleaning of pastures is proposed to be designed. Via this framework, it is possible to measure the density of weeds and empty spots through images and score the state of pasture productivity. In addition, it is possible to produce 2D weed density maps, which provide a better view of the pastures. Finally, this field of study identified a Microservices software framework to implement automatic functions in the IoT–Fog–Cloud ecosystem, which will enable the development of intelligent decision-making systems based on the IoT context [21].

Another important field is the IoT/sensor networks operational optimization. The design, implementation and evaluation of an algo-handover rhythm for wireless sensor networks (WSN) are presented where different tests were performed, and it was identified that the proposed design can reduce the energy consumption by several orders of magnitude compared to existing handover algorithms for WSNs [22]. In this same field of study in [23], an Energy Efficient and Fault-Tolerant Hierarchical Clustering Algorithm for Wireless Sensor Networks (WSN) is proposed, which the authors named (FEHCA). The simulation results are encouraging as they enable better decision making on sensing data.

As described in previous paragraphs, this research presents the development of a LoRa network architecture, which allows monitoring variables inherent to agricultural processes such as: ambient temperature, relative humidity, soil moisture and ultraviolet radiation in mountainous areas of the Andean region of Ecuador. This will enable farmers to make timely decisions regarding agricultural activities to improve production and reduce losses due to adverse climatic effects.

## 3. LoRa Overview

**Figure 1:**
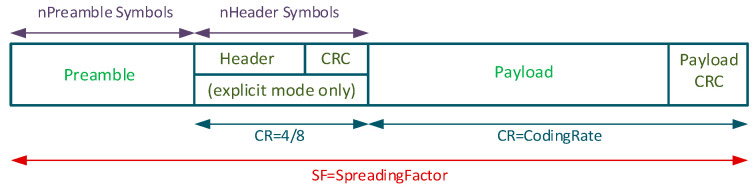
LoRa package format.

LoRa is a LoRaWAN physical layer technology, licensed free in ISM bands, characterized by low bandwidth and a limited number of messages [24,25]. It uses Chirp Spread Spectrum (CSS) modulation that allows long-range and low-power consumption [26]. In LoRa communication, some parameters can be customized, such as the spreading factor (SF), the coding rate (CR) and the bandwidth (BW). The values of each parameter depend on the region where the LoRa devices are installed. In Europe, it uses bands of 868 MHz, 915 MHz in North America and 433 MHz in Asia [25,27]. Nevertheless, the SF can vary between 7 and 12. The higher it is, the higher the signal-to-noise ratio (SNR), the sensitivity, the coverage range, the symbol time (Ts) [28] and the time on air (ToA); that is, the packet transmission time. LoRa modulation can transmit arbitrary frames using two types of packet formats, explicit and implicit. Generally, the packet structure consists of four elements: preamble, header (optional), data payload (limited to 255 bytes) and an optional payload CRC [27,29]. The format is shown in Figure 1.

A typical LoRa network operates with a bandwidth of 125 kHz, 250 kHz or 500 kHz [30]. The increase in bandwidth allows for higher transmission speed, but it becomes more susceptible to errors [31]. In Equation (1), the symbol time ($T_{s}$) is linked to the bandwidth and the spreading factor (*SF*).

(1) $$T_{s}=\frac{2^{SF}}{BW}$$

LoRa includes a forward error correcting code. The code rate (*CR*) is given by $CR=\frac{4}{4+n}$, with n∈{1,2,3,4}. Taking into account that the bits of information are transmitted by symbol. The useful bit modulation rate $R_{b}$ [32], is defined by Equation (2):

(2) $$R_{b}=SF\times \left(\frac{BW}{2^{SF}}\right)\times \left(\frac{4}{4+n}\right)$$

To calculate the time on air for the transmission of a LoRa packet [27,28,29], Equation (3) is presented:

(3) $$ToA=\frac{2^{S}F}{BW}(NP+4.25+SW+max(H,0))$$

$$H=\left[\frac{8PL-4SF+28+16CRC-20IH}{4(SF-2DE)}\right](n+4)$$

where n is the value of the parameter belonging to *CR* = 4/(4 + *n*). NP is the Number of programmable symbols of the preamble. SW is the length of the sync word. PL is the number of PHY useful payload bytes. *CRC* is *CRC* presence (1 = yes; 0 = no). IH is the presence of the PHY header (1 = no; 0 = yes) and *DE* is the use of data rate optimization (1 = enabled; 0 = disabled).

The received signal strength indicator (*RSSI*) is an important measurement parameter that reflects the quality of LoRa links [28]. With the noise factor (*NF*) of the receiver and the transmitted power P_TX, it is possible to estimate the *SNR* of the measured *RSSI* values, according to [28,32]:

(4) SNR=RSSI+174−10(BW)−NF

where NF = 6 dB, which is the noise and *RSSI* is the receiver sensitivity −137 dBm [27], *BW = 125 Khz, SF = 12* and where $R_{b}$ = 293 bps.

## 4. Proposal Development

This section explains the design of the LoRa System used for monitoring agricultural fields in the Andean region of Ecuador. The implementation of a LoRa network without using LoRaWAN is sufficient for the case study; since the network of nodes communicating through the gateway is minimal, this is low power monitoring [33]. Furthermore, LoRa represents the physical layer within a LoRaWAN network. For the coverage area, the applied system offers scalability, security, management and an affordable cost for the community population whose economic income depends solely on agricultural activities. In this context, the system will serve as a basis for the implementation of techniques such as phytosanitary control, intelligent irrigation, and production forecasting among others. The main elements are described in Table 1.

### 4.1. System Architecture

**Figure 2:**
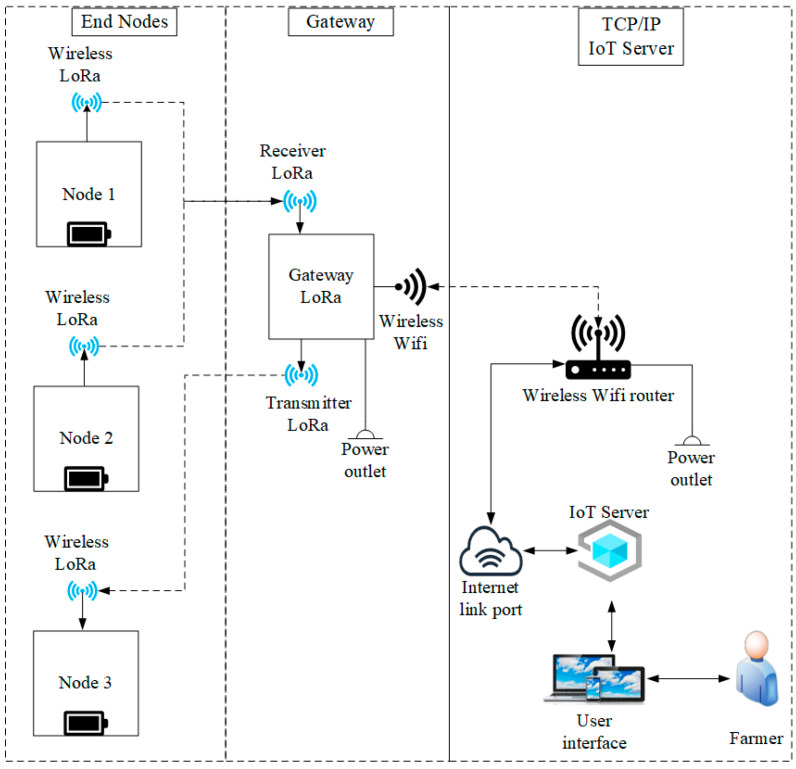
General architecture of the LoRa network system.

The Internet of Things (IoT) design based on LoRa communications is composed of different levels and elements. In Figure 2, the general architecture of the LoRa network system is presented, composed of end nodes, a gateway and an IoT server. Nodes 1 and 2 collect information from the agricultural fields by means of sensors, while node 3 is arranged for the activation of an actuator. The gateway is in charge of receiving and forwarding the information of the climatic variables coming from the final nodes to the IoT server to be visualized in a user interface that also controls the activation of a device through node 3. Therefore, in order to have a long-distance data transfer capability, the standard LoRa protocol is utilized.

### 4.2. System Description

In most cases in the Andean region of Ecuador, agricultural fields are not supervised, making it difficult to ensure adequate control of adverse weather conditions to improve crop production. In addition, the geographic areas dedicated to the cultivation of agricultural products are often difficult to access with a rocky orography and a deficit of communication infrastructure [34]. Therefore, the need to provide a monitoring and tracking system for LoRa crops is a priority and crucial not only for sustainable agriculture, but also for the protection of the micro-economies and food livelihoods of indigenous communities through controlled supervision. Therefore, a new contribution is made by introducing other prototype devices designed and created under experimental testing.

The design and fabrication of the final nodes together with the gateway are explained in detail below, and the configuration of the user interface is illustrated.

### 4.3. Final Nodes

The final nodes were custom designed with adaptability criteria capable of operating autonomously to facilitate deployment on agricultural fields and enable monitoring and control with LoRa wireless communication technology in the Andean region of Ecuador.

The electronic processor of the final nodes was a TTGO LoRa32-OLED V1 module that integrates an ESP32 microcontroller, a 0.96-inch OLED display and a LoRa module based on the SEMTECH SX1276 chip with a frequency of 868–915 MHz and a high transmission range that is very reliable [35]. Additionally, it can be programmed by Arduino IDE with some preinstalled libraries OLED, LoRa, etc.

**Figure 3:**
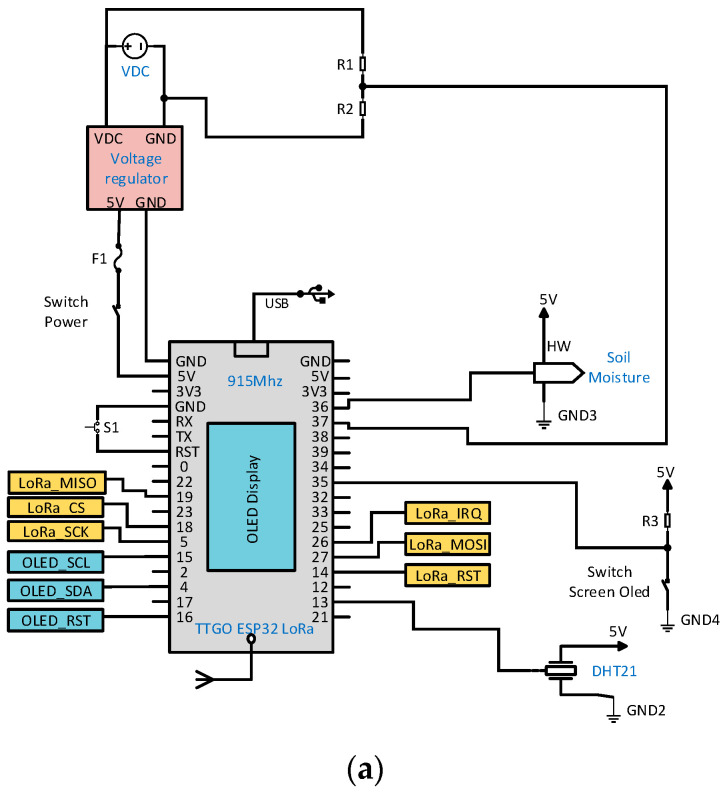

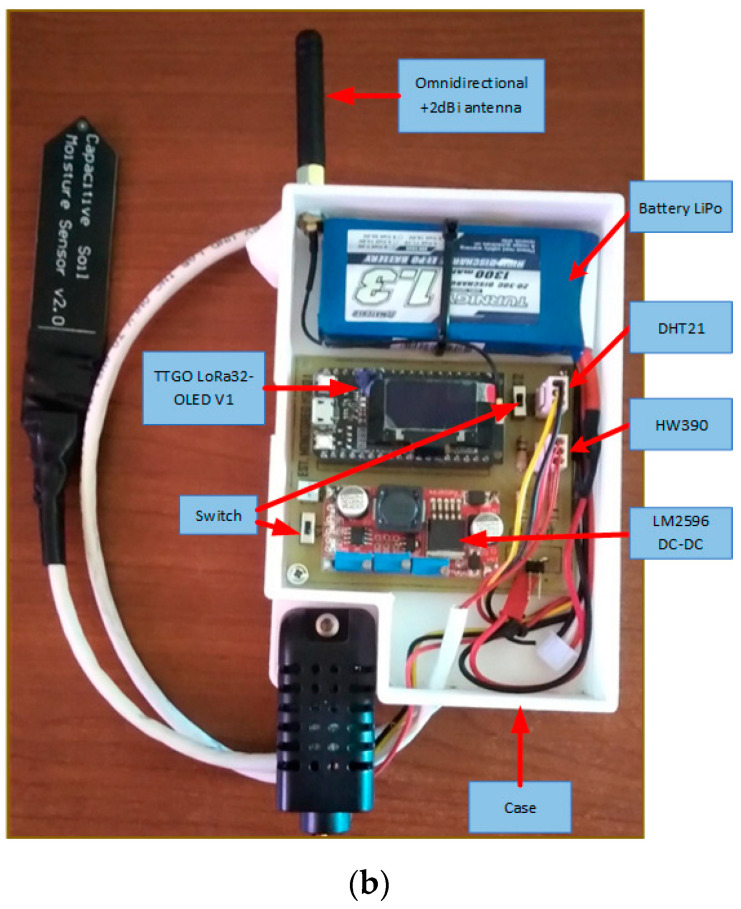
(**a**) Electronic schematic and (**b**) manufacture of transmitter node 1.

Figure 3a shows the electronic diagram of the transmitter node 1. For supplying electrical power to node 1, a rechargeable 2 Cell 1300 mAh LiPo TURNIGY battery at 7.4 V, a DC-DC LM2596 module configured to supply 5 V to the MCU, to the DHT21 sensors measuring temperature, relative humidity and to the capacitive soil moisture sensor HW390 were available.

For the synergy of the electronic components, a PCB board was designed, which is also protected by a customized IP65 internal protection grade housing 3D printed with PLA thermoplastic as shown in Figure 3b.

**Figure 4:**
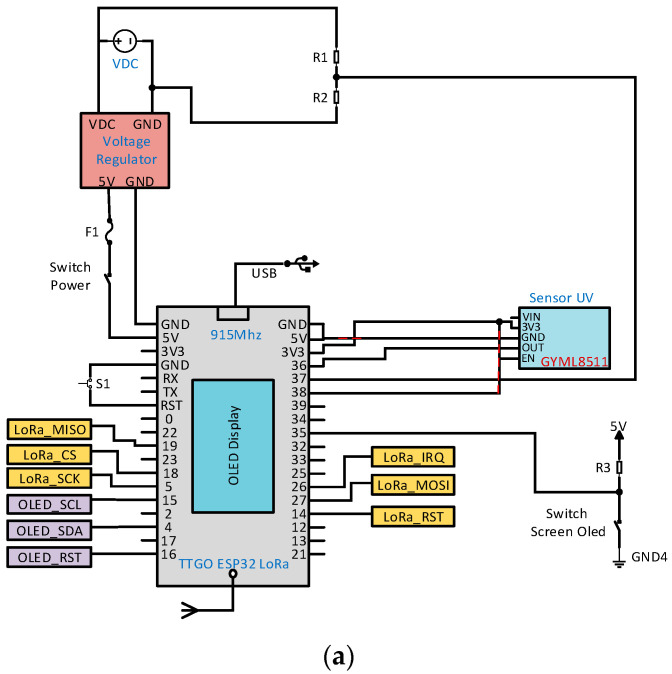

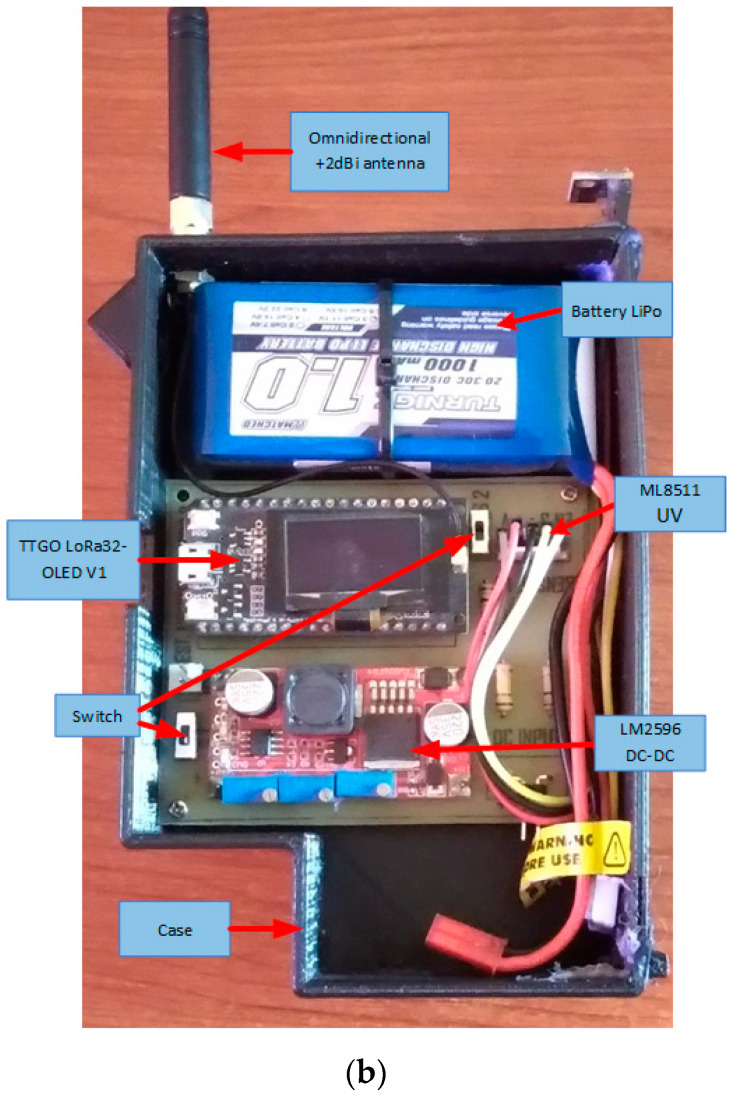
(**a**) Electronic schematic and (**b**) manufacturing of transmitter node 2.

Figure 4a shows the electronic diagram of the transmitter node 2; while Figure 4b shows the manufactured device. Unlike node 1; node 2 switches to the 3 Cell 1000 mAh LiPo TURNIGY battery at 11.1 V, which has an ML8511 analog sensor for UV radiation measurement under 5 V operation.

Receiver node 3, performs the function of receiving information from the gateway via LoRa wireless communication to control an actuator. Node 3, unlike the preceding nodes, reforms to a rechargeable 3 Cell 11.1 V 1000 mAh LiPo TURNIGY 3 Cell battery and a relay module with a controller interface for connecting AC (alternating current) powered equipment/machines, such as a water pump. The structure is similar to Figure 4.

### 4.4. Gateway

The function of the gateway is to forward and receive the information arriving from the end nodes via LoRa wireless communication to the IoT server using standard IP internet protocol [36].

**Figure 5:**
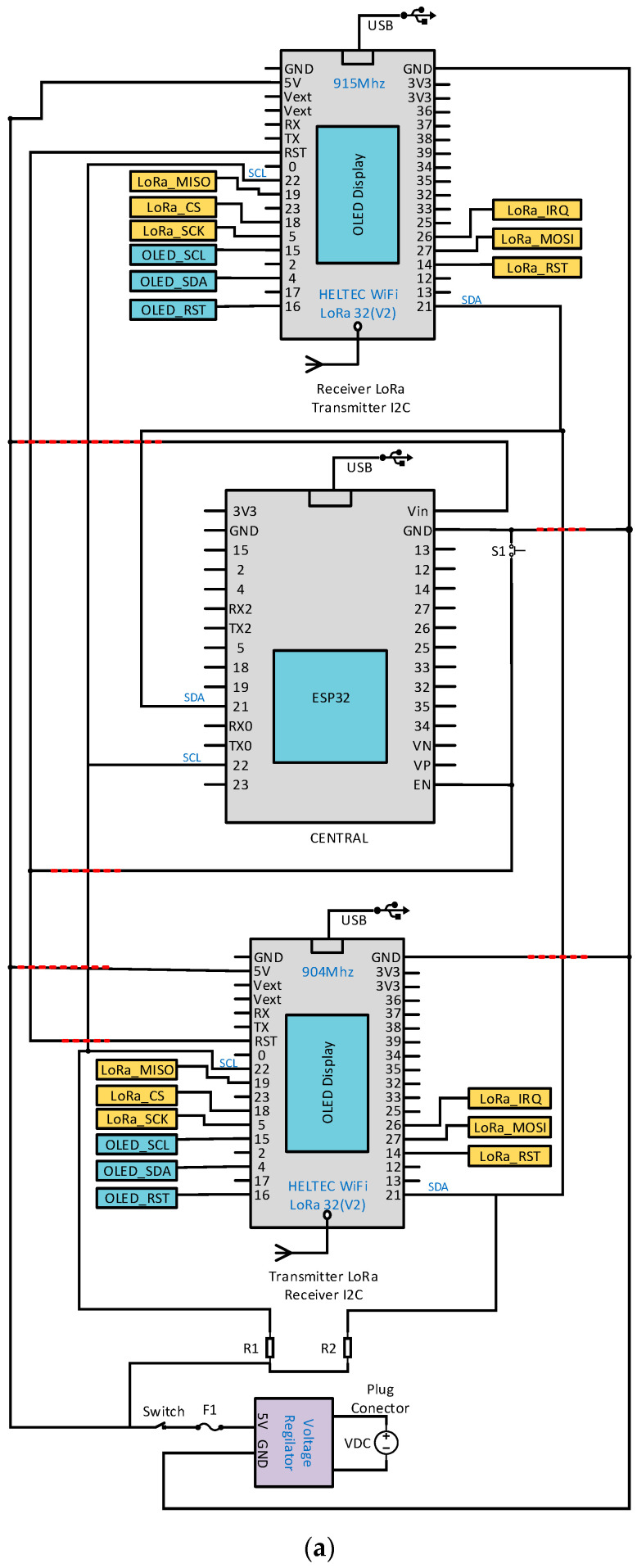

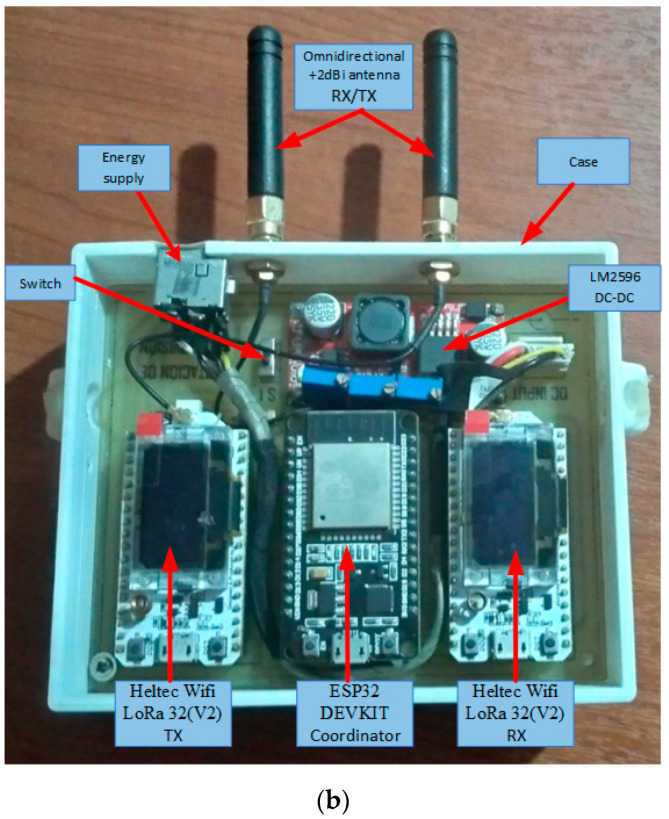
(**a**) Electronic schematic and (**b**) gateway manufacturing.

Figure 5a illustrates the electronic design of the gateway that has an ESP32 microcontroller as the central coordinator module; it is an IoT device with Wi-Fi and Bluetooth support integrated on a single development board. In the present prototype, an ESP32 DEVKIT MCU was used, which has an on-board USB serial converter and a micro-USB port for power supply, integrated 4 MB flash memory, GPIOs with PWM function, I2C, SPI, AD/DA converters, among other functions [37]. The ESP32 DEVKIT MCU allows sending and receiving information via the integrated Wi-Fi chip to and from the user interface over the IP network. For communication with the end nodes, the Heltec Wi-Fi LoRa 32(V2) module was used, which has the integrated SEMTECH SX1276 LoRa chip and the ESP32 programmable microcontroller and incorporates a 0.96-inch OLED display. The chip operates at a frequency of 902–928 MHz with a receive sensitivity of −127 dBm, an omnidirectional UHF antenna and a gain of 2 dBi [18]. With the Heltec Wi-Fi LoRa 32(V2) modules, 2 independent LoRa wireless channels were created, one for transmitting and one for receiving data. The synchronous information flow between the ESP32 DEVKIT microcontroller and the Heltec Wi-Fi LoRa 32(V2) MCUs was achieved with I2C digital communication, generating a functional gateway for LoRa Full Duplex communications. Power supply took place with the HP 120VAC charger at 1.6 A/60 Hz, the DC 18.5 V/3.5 A output, and a DC-DC LM2596 module configured to provide 5 V to the gateway MCUs.

A PCB board was designed for the synergy of the electronic components that are protected against external IP65 weather conditions with a custom-made housing printed with 3D PLA thermoplastic as presented in Figure 5b.

### 4.5. Server Communication

It is in charge of receiving and processing the data packets coming from the end nodes and administering and managing the configuration required by the network. The data are acquired by the server API; i.e., Thinger.io so that users can control the environment of their field in order to use the data log and have an action based on field monitoring [38].

### 4.6. User Application

The user interface allows data to be visualized on an IoT platform [39], through the deployment of sensor nodes. Data from agricultural fields can be collected and transmitted to control actuators. Additionally, from the user application, proper monitoring and management of agricultural production are performed [40].

**Figure 6:**
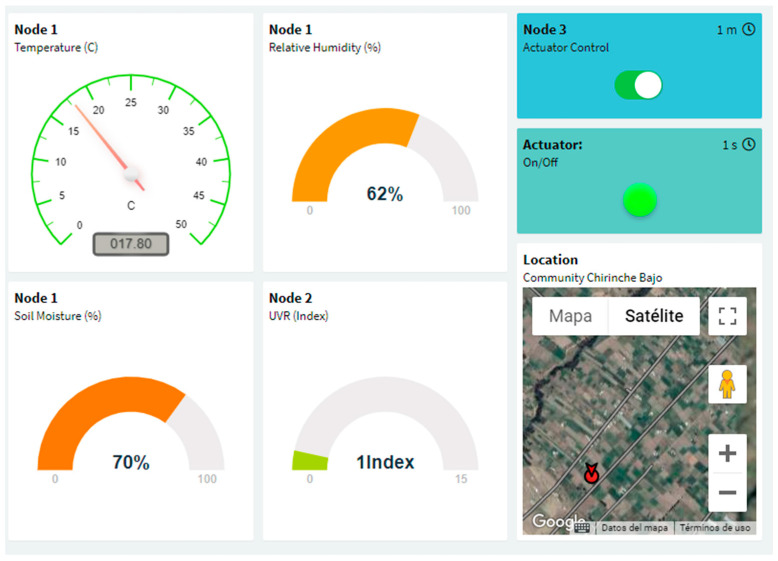
User Interface.

A graphical user interface was designed using the IoT platform Thinger.io as shown in Figure 6. The user interface allows farmers to obtain crop data and information and take control of the actuators [36]. In the interface, the value of temperature, relative humidity, soil moisture, UV radiation and battery level of the transmitter nodes can be displayed. In addition, an actuator such as a water pump can be controlled.

## 5. Testing and Experimental Configuration

To evaluate the performance of the proposed LoRa network, it was implemented and deployed in a rural area of the Andean region of Ecuador. In this Section, the deployment site and the metrics that characterize the connectivity between LoRa devices are analyzed.

### 5.1. Test Area

**Figure 7:**
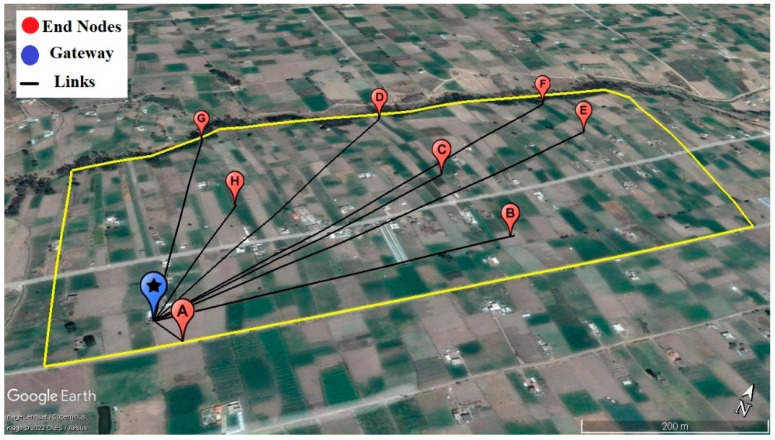
Spatial distribution of nodes and gateway on the digital surface elevation model, Chirinche Bajo community, Salcedo, Ecuador.

The environment used to evaluate the coverage of the LoRa network was in the rural community of Chirinche Bajo, Salcedo, Ecuador. The geographic location of the gateway and end nodes were deployed in an area of 50 hectares, as illustrated in Figure 7.

As is characteristic of the Andean zones, the Chirinche Bajo community has different environmental particularities such as agricultural areas with altitudes ranging from 2910 to 3040 m above sea level, slopes of 0.3% to 15%, terrain with a hard orography, irregularities in the soil and lush Eucalyptus trees in the community boundaries. Most of the people in the community are dedicated to farming potatoes, corn, onions, pasture, and caring for domestic animals such as cattle, pigs and poultry.

**Figure 8:**
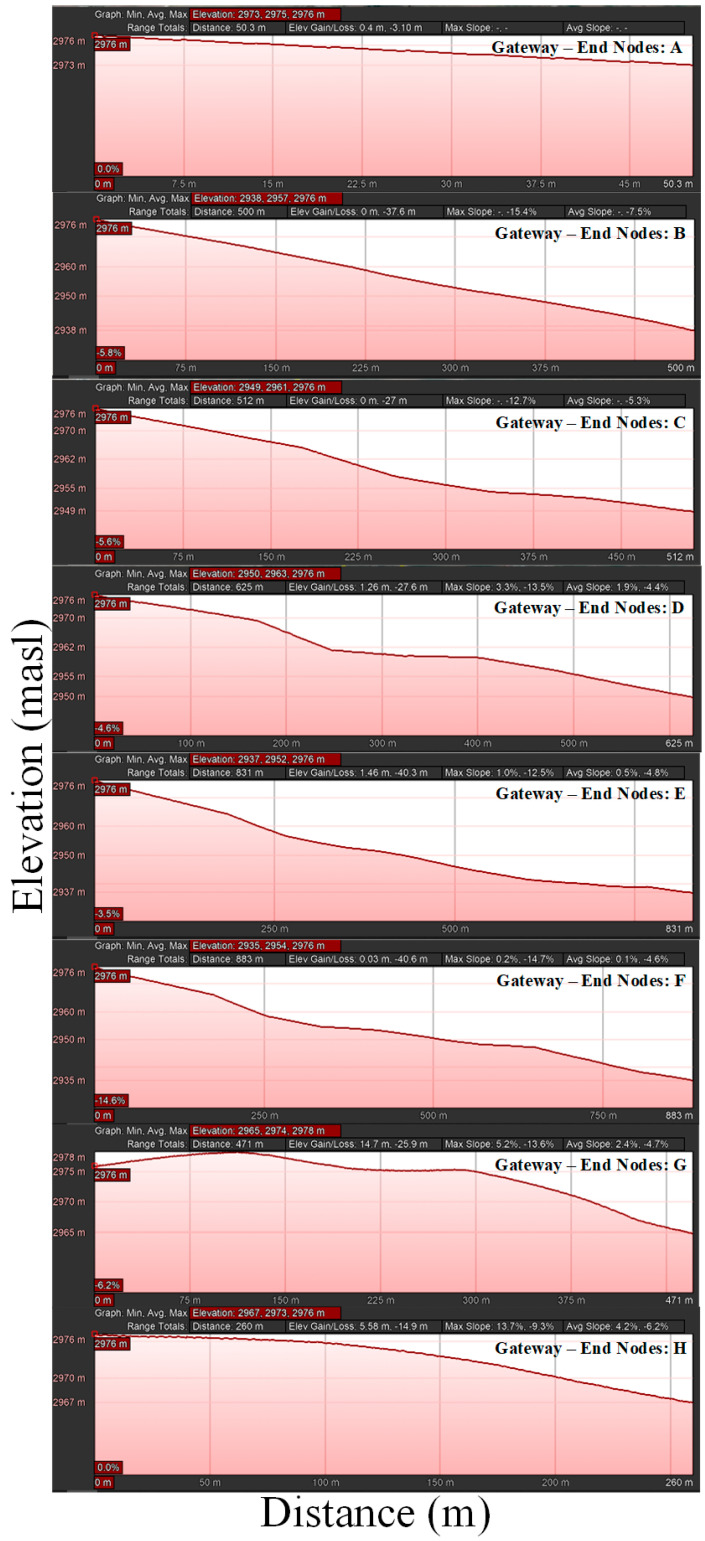
Longitudinal cross-sectional profile of link Nodes—Gateway.

To plan the deployment, it is important to obtain some insights into the communication range [41]. The satellite map in Figure 8 illustrates the position of the gateway and end nodes distinguished by blue and red points, respectively. Table 2 shows the approximate straight-line distance of the different positions from the gateway.

### 5.2. Communication between Nodes and Station

The end nodes were located in the agricultural fields of the Chirinche Bajo community, while the gateway was located in an inhabitant’s house from where it was linked to the Internet through a wireless Wi-Fi connection.

**Figure 9:**
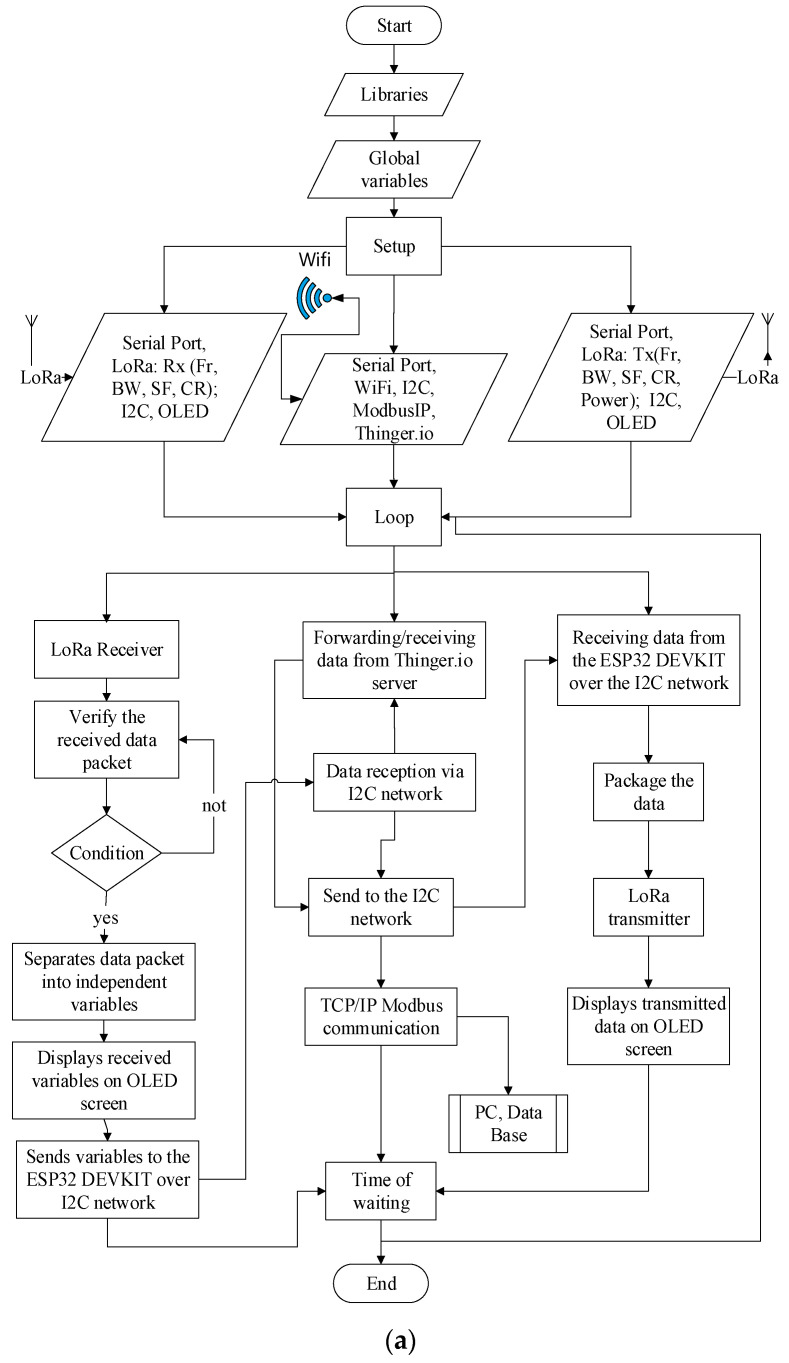

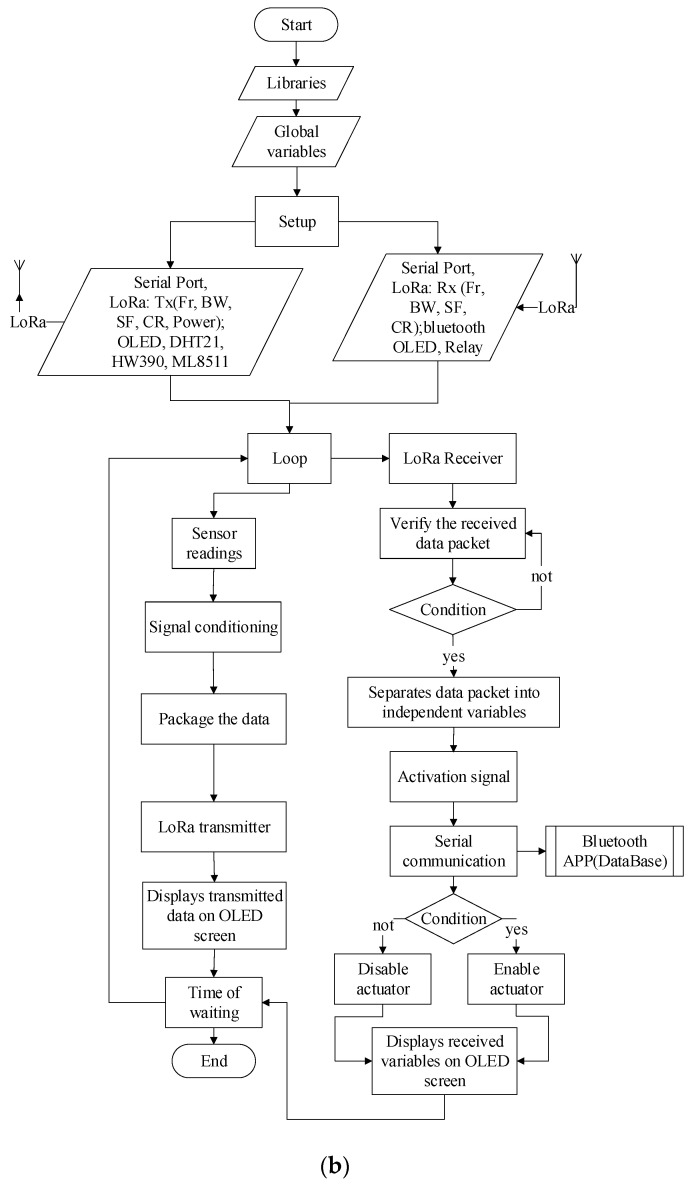
(**a**) Flowchart of the optimized gateway algorithm and (**b**) Flowchart of the optimized end-node algorithm.

Three autonomous networked end nodes were considered for the experimental test. Nodes 1 and 2, working at a frequency of 915 MHz for uplinks, while node 3 at 904 MHz for downlinks, were placed on poles 1 m above the ground, as illustrated in Figure 9a. Eight different locations were chosen based on the spatial distribution in agricultural fields and topography, in order to cover all possibilities and different types of environments, short and long distance, Line-of-Sight (LoS) and Non-Line-of-Sight (NLoS), the latter being affected by obstructions resulting from houses, trees, crops and slopes, as can be seen in Figure 8.

The LoRa gateway has 2 channels at frequencies of 915 MHz and 904 MHz for uplink and downlink by full-duplex communication. The gateway was placed on the roof of a house (1°04′58.8″ S 78°38′54.4″ W) at 4 m from the ground with the antennas placed vertically (normally a base station of this type is placed higher to achieve better coverage). In addition, the choice of the gateway location is based on two reasons. First, electrical power. As the gateway communicates with both the end nodes and the Internet, the power consumption is higher. Thus, a direct connection to the power outlet would avoid supply problems. Second, the connection to the Internet [42]. The gateway can be connected via Wi-Fi directly to the switch located in the house. Thus, communication with the IoT platform Thinger.io is ensured.

### 5.3. Experimental Configuration

Radio planning, which evaluates the performance of the LoRa network operating at 915 MHz and 904 MHz, is presented.

Based on the number of LoRa parameters that can be configured, a configuration notation is defined for the experimental tests: frequency (Fr), bandwidth (BW), spreading factor (SF) and coding rate (CR).

Nodes 1 and 2 transmit information of environmental conditions (temperature, humidity and UV radiation) to the gateway at 2000 ms intervals. The data received by the gateway were stored on a PC. Node 3 receives the payload packets from the gateway and these were sent via Bluetooth to a mobile application for storage. The study focuses on nodes sending/receiving packets with a payload length of 16 bytes, 8 bytes and 4 bytes.

The link checks at each location were performed by configuring the end node and the gateway with identical transmit/receive parameters going through various combinations for three values of spreading factor SF (7, 9 and 12), keeping a constant value in the coding rate CR (4/5) and 125 kHz in the bandwidth parameter (BW). The isotropic transmit power is set at 14 dBm, the maximum allowed [43], using antennas with 2 dBi gain. The main parameters are shown in Table 3.

The LoRa wireless communication parameters in Table 2, were customized in an optimized algorithm in C language in the Arduino IDE. The optimized algorithm addresses different parameters [44,45] pre-installed libraries such as LoRa, OLED, etc.; Fr, BW, SF, CR; variable acquisitions, reception/transmission and data flow occurring within a period of 2000 ms. The MCUs used in the research had their own optimized algorithm.

During the experiments, each end node was configured to transmit 100 packets to the LoRa gateway; as well as downstream communication node 3 received 100 payload packets.

The flowchart of the optimized gateway algorithm is illustrated in Figure 9a.

A summary of the simplified logic of the end-node operation is presented in Figure 9b.

### 5.4. Metrics

During the experiments we used different metrics to characterize the connectivity between the transmitter and receiver (See Table 4):

- The packet delivery ratio (PDR), provides information about the reliability of the communication; the PDR is calculated as the number of packets received by the gateway with respect to the total number of packets sent, with a value of 100% implying success and a value of 0 implying no success [41].
- The received signal strength (RSSI) and signal-to-noise ratio (SNR) are two physical layer PHY-level indicators of LoRa available on-chip, which we use to characterize the signal quality [46].
- The connectivity range represents the measured distance between the receiver and the transmitter. Our objective was to study the connectivity range in the agricultural fields of the Chirinche Bajo community.

## 6. Experimental Results and Analysis

The metrics considered in the study were packet delivery ratio (PDR), received signal strength indicator (RSSI), signal to noise ratio (SNR) and time on air (ToA), which are the parameters chosen to assess the performance of the LoRa communication uplink and downlink in the different environments of the Chirinche Bajo community. The experiments were conducted during weather variations with sun and rain from 5–23 June 2022.

**Figure 10:**
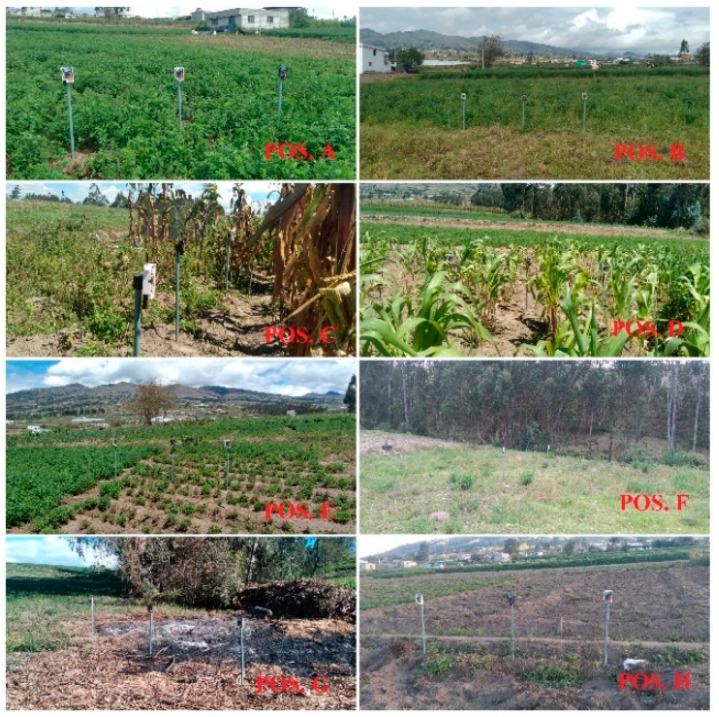
End nodes in test positions.

The LoRa gateway was placed in a fixed position during all measurements, while the end nodes were placed in different locations as illustrated in Figure 10. The evaluation was segmented into three groups: the first expedition was in the morning hours, the LoRa network was configured with SF at seven, a cloudy day with showers and a breeze was present; the average temperature was 16 °C, the relative humidity was in the range of 74–86% and the average UV radiation was in two indexes.

In the second evaluation, the SF was parameterized at nine and the weather was partially cloudy. At the end of the evaluation (POS G and H), the sun was present, the wind blew with medium intensity; the average temperature was 12 °C, the relative humidity was in a range of 74–89% and the average ultraviolet radiation was in one index. Finally, on the third excursion, it was a sunny day with high-intensity winds, the average temperature was 19 °C, the relative humidity was in a range of 53–68% and the average ultraviolet radiation was in four indices. This evaluation was carried out from 16h00.

### 6.1. PDR Package Delivery Ratio

**Figure 11:**
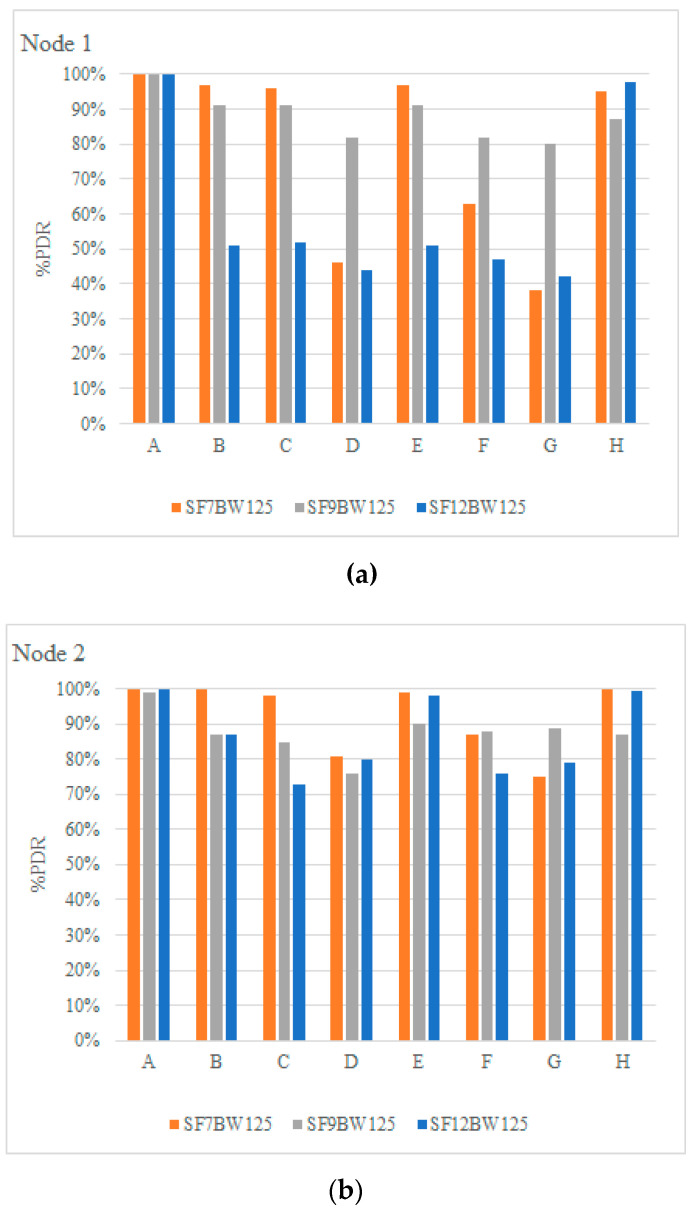
PDR: (**a**) Nodes 1, (**b**) Node 2.

In this subsection, we present the PDR results for uplink communications, i.e., the end nodes send 100 packets to the gateway with a payload length of 16 bytes (node 1) and 8 bytes (node 2) with a frequency of 2000 ms, at various distances with different SF configurations, including SF = 7, 9 and 12. Figure 11a illustrates the PDR for node 1 and Figure 11b the result for node 2. It can be visualized that the PDR decreases with increasing payload length, demonstrating an impact of packet length on frame reception. Being in the SF configuration in 12 with a PDR in the range of 42–47% with PL of 16 bytes in positions D, F and G. Furthermore, it is determined that the PDR success rate is different for each end node depending on the test environment; therefore, in position A, B, C, E and H, a better PDR is obtained than in positions D, F and G. The effect is due to the fact that positions D, F and G are located in areas with slopes of about 15%, surrounded by eucalyptus trees, vegetation and no line of sight (NLoS), thus generating more interference, more path loss and noise to the LoRa radio communication signal.

In contrast to the results obtained in [46], the tests carried out in a forested area at 90 m, there was no communication for the transmission powers tested (7, 13 and 14 dBm); in comparison with the present work, communication was achieved for this type of orography; obtaining a PDR of 42–47% in uplinks and downlinks a PDR greater than 87%, for forested areas.

**Figure 12:**
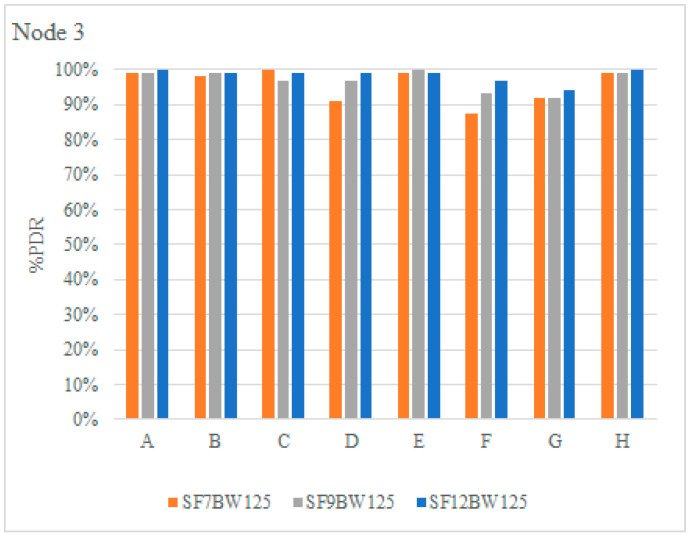
PDR Nodes 3.

Node 3 was set up for downlink communications, i.e., the gateway sends 100 packets with a payload length of 4 bytes to the end node. Figure 12 shows the PDR of node 3. It is determined that the PDR with SF of 7, 9 and 12 at positions D, F and G maintains an acceptable success rate, since they are the locations with the highest interference, a PDR of no less than 87% is displayed. It is important to note that the objective of the tests is to check the coverage of the LoRa physical layer using different propagation factors.

### 6.2. Receiver Sensitivity

Since there are many models and evaluations of the propagation of LoRa radio signals in various environments [28], this experiment focuses on testing the performance of LoRa receivers in the Chirinche Bajo community, as there are no studies conducted in these areas. The received signal strength indicators (RSSI) of the packets and the signal-to-noise ratio (SNR) were recorded at each of the positions.

**Figure 13:**
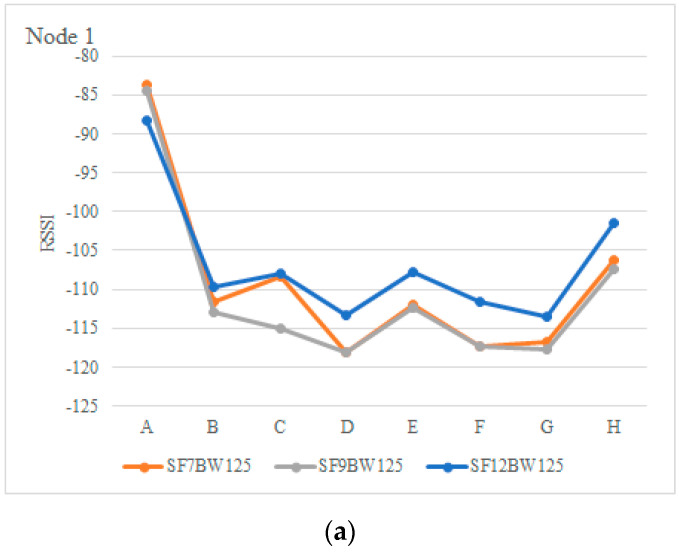

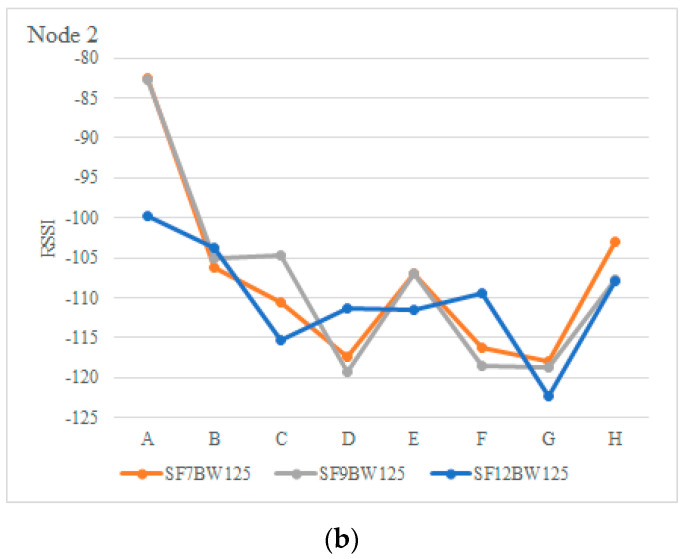
RSSI of the radio (dBm): (**a**) Node 1, (**b**) Node 2.

Figure 13a,b show the RSSI values received at the gateway from node 1 and 2 with different SFs. RSSI values around the −111 dBm and −122 dBm mark are observed at positions D, F and G. The configuration with SF equal to nine emerges as the best option to achieve the highest throughput with the lowest packet on-air time at locations D, F and G. However, the alternative (SF at 12) could also be used. This would depend on the application’s tolerance to packet loss.

**Figure 14:**
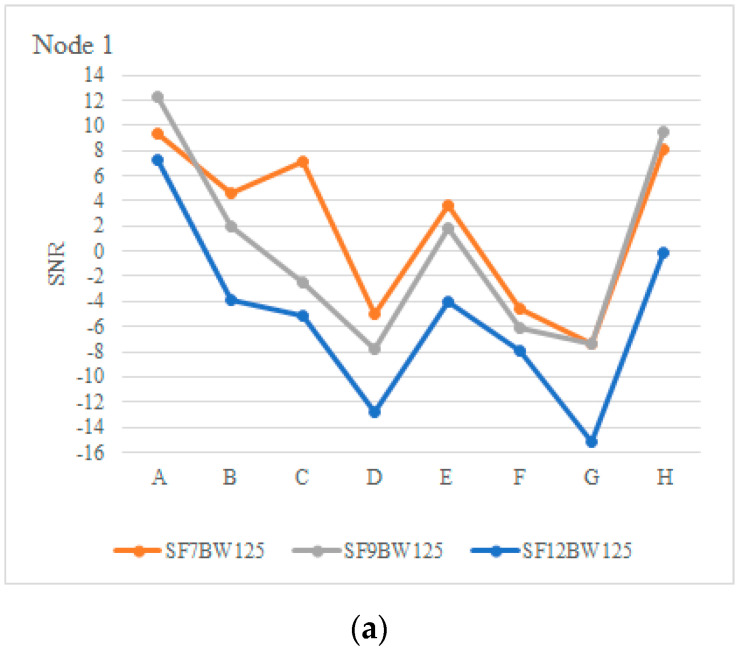

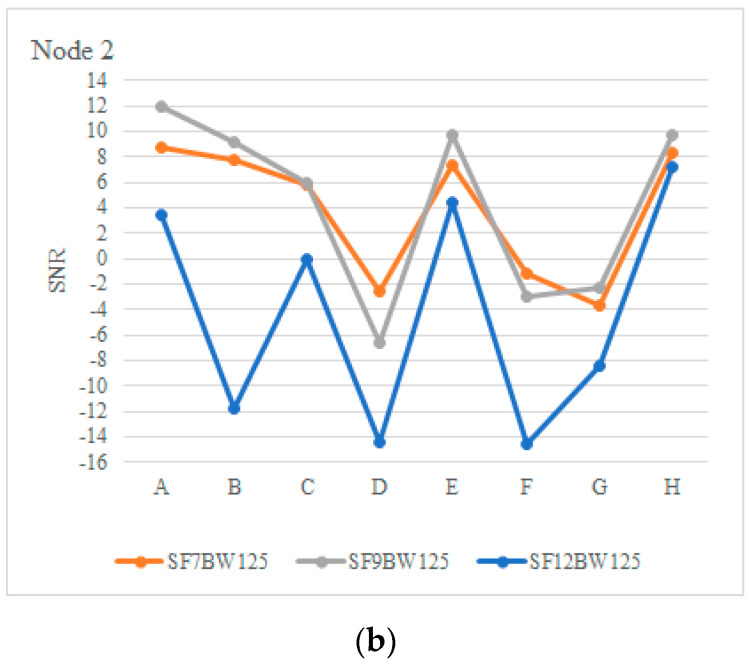
Radio SNR (dBm): (**a**) Node, (**b**) Node 2.

The signal-to-noise ratio (SNR) with the RSSI are related, since, in positions D, F and G, there is more interference in the signal flow with SNR values in a range of −2.55 and −15.14, as can be seen in Figure 14a,b. It should be noted that the signal sensitivity is largely influenced by the evaluation environment, which, in the case of positions D, F and G, was located on a 15% slope surrounded by eucalyptus trees and without a line of sight (NLoS).

**Figure 15:**
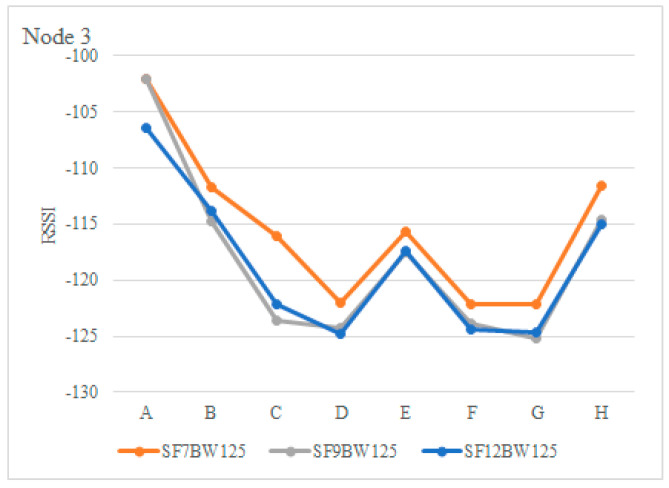
RSSI of the radio (dBm) of node 3.

**Figure 16:**
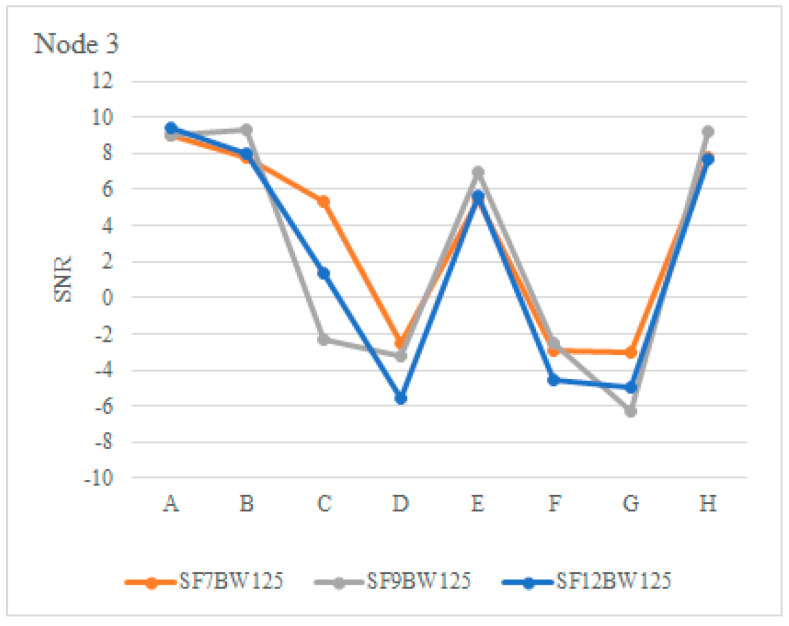
SNR of node 3.

The RSSI was evaluated in downlink communication, with a payload of 4 bytes, the sensitivity was in the range of −122 dBm and −125 dBm with SNR in a range of −2.51 and −6.27 in positions D, F and G; although, they are lower than in the uplinks, the PDR had higher efficiency. Figure 15 illustrates the RSSI of node 3 and Figure 16 shows the SNR with SF of 7, 9 and 12.

### 6.3. Time in the Air (ToA)

It is noticeable that higher BW bandwidth gives a higher data rate [37], with shorter transmission time, as Equation (2) demonstrates. However, higher BW degrades the receiver sensitivity, as revealed by Equation (4), due to additional noise integration. For the transmission of a LoRa packet, its structure comprises three elements: preamble, header (optional) and payload.

There are two types of LoRa packet format modes, explicit and implicit; where the header is removed from the packet, both the CR and payload size are fixed and must be manually configured on both sides of the radio link. Therefore, the implicit header mode reduces the transmission time of the packet, which is known in the LoRa literature as ToA calculated using Equation (3). For the study, we worked with the LoRa node parameters in Table 2.

**Figure 17:**
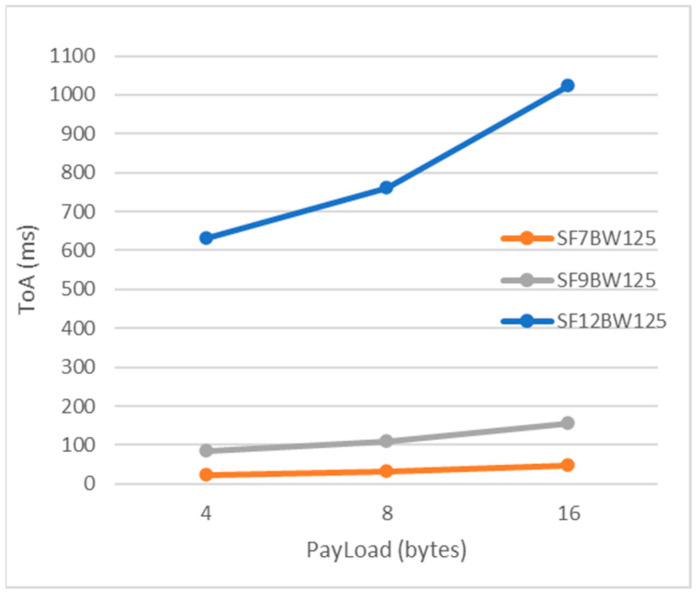
LoRa data packet transmission time.

The packet structure in transmission consisted of the preamble plus the payload of 16 bytes, 8 bytes and 4 bytes with different SF. The result showed that the higher the SF, the airtime of a packet increases. These results can be seen in Figure 17.

### 6.4. Discussion

In the literature, we identified works that have developed similar proposals. Many of the articles that were analyzed seem to assume that the terrain orography does not have much importance in the implementation of the network; these studies are focused on greenhouses. In [2,34,36,38,47], the type of orography of the site is not detailed. The different authors propose novel systems of intelligent agricultural management and monitoring based on LoRa, without considering the orography of the terrain in mountainous areas.

Another important factor that is not taken into account when applying the network is the altitude of the locations, since these studies are located below 1700 m above sea level, being a factor that affects the transmission of data packets according to [46]. The distance between the gateway and the growing site of a LoRa network in rural areas according to [38] is 700 m; reaching a spreading factor of seven at 400 m and a spreading factor of nine reaching 500 m, respectively. In this context, it is explained that as the propagation factor increases, which is the communication delay between the client and the servers, an increase in the communication delay was obtained. The proposed system in Andean areas reduces this problem by maintaining optimal communication with SF settings of seven and nine in different weather conditions as explained in Section 6. According to the results found in [2] where they evaluate the performance of a LoRaWAN network, they confirm that the performance of the network is influenced by the sending of messages with or without acknowledgement, the size of the network, the increase in the number of gateways and the distance of placement of the gateway nodes, not to mention the weather conditions and even more the terrain orography. Therefore, the study focuses on the influence of the packet transmission interval together with the number of nodes on the packet delivery rate.

Therefore, the importance of this study lies in the implementation of a monitoring system based on the LoRa network in mountainous areas of Ecuador.

## 7. Conclusions and Future Works

In the literature, there are studies on the radio range of LoRa in geographic areas that do not exceed 1700 m.a.s.l., in which it is stated that the coverage depends on the environment in which the network is implemented. So, this research was conducted, since there are no previous studies that give a detailed explanation of the LoRa network in mountainous areas above 2910 m.a.s.l. in the Andean region of Ecuador.

The orography impaired the radio-link sensitivity, which was attenuated when located in positions with slopes of 15% with the presence of eucalyptus trees in the environment and without a line of sight with respect to the Gateway. The objective of implementing a monitoring system of climatic variables for the agricultural fields of the community of Chirinche Bajo was achieved.

In Ecuador, the use of these LoRa networks is very limited due to the technological gap that exists in the Andean areas of Ecuador, in addition to the fact that agricultural processes are not technical due to the low technological knowledge of the population, which hinders the successful implementation of new technologies.

To evaluate the range and avoid data loss in LoRa radio communication, the propagation factor (SF) was set to 7, 9 and 12, keeping the CR constant at (4/5) and BW at 125 kHz, in order to assess the sensitivity and integrity in the delivery of data packets. The SF setting equal to nine, emerged as the best option to achieve the highest throughput with the shortest packet on-air time. However, the alternative (FS at 12) could also be used to achieve higher coverage. Although, this would depend on the application’s tolerance to packet loss. The performance of LoRa communication with an SF of nine achieves a packet delivery success rate (PDR) for uplinks no lower than 76% and a PDR for downlinks higher than 92%.

The scope of the LoRa network architecture is based on tests conducted in a 50-hectare field with a maximum range length of 875 m without a line of sight, obtaining adequate data delivery. The present work benefited 80 families in the community of Chirinche Bajo in the province of Cotopaxi in Ecuador, through the timely monitoring of climatic variables that mitigate agricultural losses due to adverse climatic effects.

To increase the autonomy time of the nodes deployed in the crops, a feasibility analysis should be conducted to implement photovoltaic power generators, recharge the batteries and increase the operating time.

Currently, work is being undertaken to improve security aspects by means of encryption algorithms such as Advanced Encryption Standard (AES) at the moment of transmitting the information.

In addition, according to the findings of this work, we intend to implement an automated system that allows frost control, a harmful condition that affects agricultural production in the area.

## Figures and Tables

**Table 1:** Hardware and Software Components.

	Microcontroller	Module or Sensor	Battery	Various
**Hardware**	TTGO LoRa32-OLED V1	DHT21	LiPo TURNIGY 1300 mAh, 2 Cell	DC-DC LM2596
	ESP32 DEVKIT	HW390	LiPo TURNIGY 1000 mAh, 3	Relay
	Heltec Wifi LoRa 32(V2)	ML8511	LiPo TURNIGY 1000 mAh, 3 Cell	IP65 case
	**IoT Platform**		**Programming platform**	
**Software**	Thinger.io		IDE Arduino	

**Table 2:** Node positions and distance (approximate in a straight line) from the gateway.

Position	Distance (m)
Pos A	50 m
Pos B	496 m
Pos C	508 m
Pos D	619 m
Pos E	821 m
Pos F	875 m
Pos G	465 m
Pos H	254 m

**Table 3:** LoRa communication parameters.

Parameters	Value/Configuration		
	Node 1	Node 2	Node 3
**Transmission**	Uplink	Uplink	Downlink
**Frequency**	915 MHz	915 MHz	904 MHz
**Preamble**	8 Symbol	8 Symbol	8 Symbol
**Transmission power**	14 dBm	14 dBm	14 dBm
**Coding rate**	4/5	4/5	4/5
**Propagation factor**	7, 9, 12	7, 9, 12	7, 9, 12
**Bandwidth**	125 kHz	125 kHz	125 kHz
**Package size**	16 bytes	8 bytes	4 byte
**Antenna gain**	2 dBi	2 dBi	2 dBi

**Table 4:** Summary of evaluated metrics.

Metrics	Units	Meaning
PDR	%	Package Delivery Ratio
RSSI	dbm	Received Signal Strength Indicators
SNR	db	The signal-to-noise ratio
ToA	ms	Time on Air

## Data Availability

Not applicable.
